# Referring physicians' intention to use hospital report cards for hospital referral purposes in the presence or absence of patient-reported outcomes: a randomized trial

**DOI:** 10.1007/s10198-023-01587-6

**Published:** 2023-04-13

**Authors:** Martin Emmert, Anja Schindler, Laura Heppe, Uwe Sander, Christiane Patzelt, Michael Lauerer, Eckhard Nagel, Cornelia Frömke, Oliver Schöffski, Cordula Drach

**Affiliations:** 1 Faculty of Law, Business and Economics, Institute for Healthcare Management and Health Sciences, University of Bayreuth, Prieserstraße 2, 95444 Bayreuth, Germany; 2 School of Business and Economics, Chair of Health Care Management, Friedrich-Alexander-University of Erlangen-Nuremberg, Lange Gasse 20, 90403 Nuremberg, Germany; 3 Department of Information and Communication, Faculty for Media, Information and Design, University of Applied Sciences and Arts, Hannover, Germany

**Keywords:** Public reporting, Hospital referrals, Patient-reported outcomes, UTAUT, Hospital report cards, I12

## Abstract

**Purpose:**

This study aims to determine the intention to use hospital report cards (HRCs) for hospital referral purposes in the presence or absence of patient-reported outcomes (PROs) as well as to explore the relevance of publicly available hospital performance information from the perspective of referring physicians.

**Methods:**

We identified the most relevant information for hospital referral purposes based on a literature review and qualitative research. Primary survey data were collected (May–June 2021) on a sample of 591 referring orthopedists in Germany and analyzed using structural equation modeling. Participating orthopedists were recruited using a sequential mixed-mode strategy and randomly allocated to work with HRCs in the presence (intervention) or absence (control) of PROs.

**Results:**

Overall, 420 orthopedists (mean age 53.48, SD 8.04) were included in the analysis. The presence of PROs on HRCs was not associated with an increased intention to use HRCs (*p* = 0.316). Performance expectancy was shown to be the most important determinant for using HRCs (path coefficient: 0.387, *p* < .001). However, referring physicians have doubts as to whether HRCs can help them. We identified “complication rate” and “the number of cases treated” as most important for the hospital referral decision making; PROs were rated slightly less important.

**Conclusions:**

This study underpins the purpose of HRCs, namely to support referring physicians in searching for a hospital. Nevertheless, only a minority would support the use of HRCs for the next hospital search in its current form. We showed that presenting relevant information on HRCs did not increase their use intention.

**Supplementary Information:**

The online version contains supplementary material available at 10.1007/s10198-023-01587-6.

## Introduction

The objective of publicly reporting hospital-quality performance measures is to improve healthcare quality by both stimulating quality improvement on the provider level (“Change”) and also by helping patients, purchasers, regulators, consumers, contractors, referring clinicians, and other consumers to select the “right” provider (“Selection”) [1]. First, the “change” pathway describes a mechanism by which health care providers (i.e., hospitals, nursing homes, physician practices) are motivated to improve their quality of care [1]. According to the results of latest systematic reviews, it seems that public reporting in general may stimulate quality improvement activity by means of offering new services, changing policies, change in personnel, and an increase in quality improvement activities [2–6]. Regarding the second pathway (“selection”), health care services are shifted to caregivers who provide better quality of care. Regarding both pathways, publicly available Internet rating websites [referred to in this paper as hospital report cards (HRCs)] have been developed and implemented in many high-income countries [7, 8]. So far, patients, their families, and prospective patients (i.e., all of us) are seen as the primary target of most public reporting initiatives [9]. Most evidence has been conducted to investigate whether patients use publicly reported quality information to search for and select healthcare providers [10–12]; so far, with limited success at best [2, 6, 13]. However, less information is available regarding whether publicly available quality information plays a role from the physicians’ perspectives in referring patients to hospitals [14, 15]. This seems to be surprising since most patients trust in their referring physicians’ recommendation regarding what hospital to choose [15]. Therefore, referring physicians in particular seem to be one major target group of HRCs so as to direct patients to well-performing hospitals [1, 14].

In Germany, the indication for hospital treatment comes from outpatient-based physicians (both general practitioner and specialists). Thereby, the referring physician may specify the two nearest reachable suitable hospitals on the referral but is not obliged to do so; however, the referring physicians’ recommendation is not binding. The patient may either choose to follow this recommendation or is free to choose any other hospital [16, 17]. The only prerequisite for the statutory health insurance (SHI) fund to cover the costs is that the selected hospital is approved for the provision of treatment to SHI patients (e.g., University hospitals, hospitals that are included in the state's hospital plan) [18, 19]. [It is also important to mention, that hospital referrals in the German health care sector are made to a specific hospital rather than to a specific doctor within this hospital.] Besides this, patients can directly access and choose hospitals via after-hours care and emergency care [20]. Following the patients’ free choice among hospitals (see above), publicly reported quality information about hospitals in Germany—and HRCs in particular—might have a larger impact on hospital choice than in countries where the patient`s choice is more limited (e.g., United States, Bolivia, Colombia) [20].

The available literature has presented a limited impact of publicly reported quality information on the hospital referral behavior of physicians. For example, first surveys of cardiologists in Pennsylvania in 1996 [21] and New York in 1997 [22] have demonstrated that even though most cardiologists were aware of cardiac surgery report cards, their impact on hospital referral behavior was limited. More recently, Brown and colleagues (2013) have shown that only 25% of surveyed cardiologists in New York have reported a moderate or substantial influence on referral decisions [23]. Evidence from the Netherlands (2014) and France (2016) and have demonstrated even lower numbers; here, 12% resp. 14% of physicians had used comparative performance information when selecting a hospital [24, 25]. More recent evidence from Germany (2017) has shown that every fifth referring physician (21%) stated that he/she had been influenced by the Nuremberg Hospital Quality Reporting System (NHQRS) [14].

The literature has tried to gain a better understanding of why public reporting instruments have not been more successful and what improvements could be made. For example, the analysis of the German NHQRS showed that referring physicians criticized the underlying data, the design of the ranking, the omission of important quality information (e.g., Patient-Reported Outcomes), or methodological issues [14]. Other studies reported weaknesses in report card content, design, and accessibility as possible reasons [26]. Regarding the content of public reporting instruments, one promising strategy to increase their impact is to give more weight to Patient-Reported Outcomes (PROs) [26, 27]; that is, to report on “any aspect of a patient’s health status that comes directly from the patient.”[28] In general, PROs refer to feedback from patients about their health and functional status (mostly before and after surgery), measured on a quantified scale using generic or condition-specific instruments [29]. While PROs were developed for use in research initially, more recently they have been used to assess and compare the outcomes achieved by healthcare providers [30]. Nevertheless, there is no study available that investigates whether the presence of PROs on HRCs might have an effect on the hospital selection behavior of referring physicians.

In sum, there is a lack of studies exploring the reasons for the low HRC adoption. Therefore, this article presents an in-depth study exploring the adoption of HRCs among referring physicians. To this end, we apply a theoretical framework that is well established in the Information Systems discipline, namely the Unified Theory of Acceptance and Use of Technology (UTAUT) [31]. Recently, UTAUT has been gaining importance in health-related contexts in general [32], and was also used to identify factors that explain the use of HRCs among current users and non-users in the US [33] as well as among patients in Germany [34]. In this context, the present study also explores the first time the specific role of Patient-Reported Outcomes, which have attracted attention in both literature and practice recently from the perspective of referring physicians.[26, 27, 35–37]. So far, it remains unclear whether and to what extent referring physicians value PROs when selecting a hospital for referral purposes.

Therefore, the objective of this study is to address this research gap by answering the following four research questions (RQ): (RQ 1) Does the presentation of PROs on HRCs have an impact on the intention to use HRCs for hospital referral purposes? (RQ 2) What are the determinants influencing referring physicians to use HRCs? (RQ 3) What importance do both traditional quality measures as well as PROs have for hospital referral purposes from the perspective of referring physicians? (RQ 4) What do orthopedists from the German outpatient sector think of using HRCs for referral purposes?

## Methods

This study used a mixed methods approach. After performing a systematic literature review, we conducted qualitative research methods (i.e., semi-structured interviews) to identify the most important quality measures for choosing a hospital for hip replacement surgery from the perspective of referring physicians. Based on this, the quantitative part of this study was developed to learn more about referring physicians’ intention to use hospital report cards (HRCs) in the presence or absence of Patient-Reported Outcomes (PROs).

### Preliminary literature review and qualitative steps

In a first step, we conducted a systematic search procedure on Medline (via PubMed) and the Cochrane Library to detect studies that aimed to identify relevant criteria for referring patients into hospitals for hip replacement surgery from the perspective of referring physicians. The search was carried out in September 2020 and aimed at identifying English and German language literature published since 2010. The review complied with the Guideline from the Cochrane Collaboration [38]. Our search strategy was based on previously published literature and slightly modified [39]. As a result, 2,246 potentially relevant papers were identified in the electronic databases. After eliminating duplicates and judging titles and abstracts in a first step as well as full papers in a second step, 16 studies were considered relevant (see Supplementary Material 1 for the PRISMA 2020 flow diagram). In sum, 39 criteria were derived from those studies. In the next step, we excluded 20 criteria that are not publicly available in the German hospital sector for public reporting purposes (e.g., waiting times, MRSA events), so that 19 criteria remained for the next step (see Supplementary Material 2 for a brief overview).

Afterwards, we conducted semi-structured interviews (November 2020 to January 2021) with 15 referring orthopedists (mean age 56 years, 20% female), who were invited via mail to participate in this study. Written informed consent was obtained from all participants. Participating orthopedists received a short survey before conducting the semi-structured interviews to learn more about the hospital referral decision-making and the importance of the 19 criteria (see above). Within the semi-structured interviews, we explored the stated responses in more detail and determined the most relevant criteria. Based on both steps, we derived the following six attributes that were of major importance for selecting a hospital from the referring physicians’ perspective: the number of cases treated; certification as an EndoProstheticsCenter; the rate of postoperative complication rate; the rate of confirmed diagnosis prior the surgery; the 1-year revision surgery rate; and the rate of mobility at hospital discharge. Individuals who completed the qualitative study received 50 euros.

### Research model for the quantitative part of the study

As stated above, the research model for the quantitative part of the study draws on the Unified Theory of Acceptance and Use of Technology (UTAUT).[31]. UTAUT integrates core elements of eight models of IT acceptance and use (e.g., Technology Acceptance Model) and was shown to outperform each individual model in terms of explanatory power [31]. Recently, UTAUT was also used to identify factors that explain the use of HRCs among current users and non-users in the US [33] as well as among patients in Germany [34]. Based on the latter studies and further literature [31, 33, 40], we adjusted the research model to the context of our study and adapted the wording of the original UTAUT items to the context of using HRCs for hospital referral decision-making. [While Supplementary Material 3 presents the research model, Table 1 gives an overview of the constructs and measurement items.]

**Table 1:** Constructs and measurement items [study sample (*N* = 420)]

Construct	ID	Item	Loadings
Attitude (AT)	AT1	Using hospital report cards for referring patients into a hospital is a good idea	0.822
	AT2	Hospital report cards makes searching for a hospital more interesting	0.797
	AT3	I like working with hospital report cards	0.864
	AT4	Working with hospital report cards is fun	0.767
	AT5	I am open for using hospital report cards to search for a hospital	0.822
Effort expectancy (EE)	EE1	My interaction with hospital report cards is clear and understandable	0.802
	EE2	It is easy for me to become skillful at using hospital report cards	0.819
	EE3	Learning how to use hospital report cards is easy for me	0.758
	EE4	I find hospital report cards easy to use	0.823
Facilitating conditions (FC)	FC1	I have the resources necessary to use hospital report cards (e.g., internet connection, home computer).*	–
	FC2	I have the knowledge necessary to use hospital report cards.*	–
	FC3	Hospital report cards are compatible with other information sources (e.g., opinion of colleagues) that I use to search for a hospital	0.910
	FC4	I think that using hospital report cards fits well with the way I like to search for a hospital	0.931
Performance expectancy (PE)	PE1	I find hospital report cards useful to search for a hospital for my patients	0.905
	PE2	Using hospital report cards enables me to search for a hospital more quickly	0.895
	PE3	Using hospital report cards helps me find the best hospital	0.923
	PE4	If I use hospital report cards, I will increase the chances of my patients of feeling well after the surgery	0.882
Social influence (SI)	SI1	People who influence my behaviour think that I should use hospital report cards	0.850
	SI2	People who are important to me think that I should use hospital report cards	0.867
	SI3	I would feel uncomfortable if my colleagues use hospital report cards and I do not	0.881
	SI4	I would feel uncomfortable if my patients use hospital report cards and I do not	0.869
Intention to use (IU)	IU1	I intend to use hospital report cards for the next hospital referral of my patients	1.000

All items adapted from Venkatesh et al. (2003)

*Based on the instrument validation process, item was removed

The dependent variable is the intention to use HRC (“I intend to use hospital report cards for the next hospital referral”), which was shown to be a strong predictor of actual use behavior [31]. In addition to four originally proposed main independent variables [(1) effort expectancy; i.e., the degree of ease associated with the use of HRC; (2) facilitating conditions; i.e., the degree to which an individual believes that an organizational and technical infrastructure exists to support the use of HRCs; (3) performance expectancy; i.e., the degree to which an individual believes that using HRC will help him or her to attain performance gains; and (4) social influence; i.e., the degree to which an individual perceives that important others believe he or she should use HRC], we added attitude (i.e., the degree to which an individual has positive or negative feelings about using HRC [41]) as a fifth independent variable to the research model [33, 42]. As suggested, we used age, gender, and experience as moderating variables [31, 34] and existing hospital affiliations (i.e., whether the referring physician has an affiliation and performs the surgery herself/himself) as well as hospital-quality perceptions (i.e., whether an individual perceives that there are no, small, or big quality differences among hospitals) as control variables [33].

### The survey instrument and survey sample

The survey instrument consisted of four parts. Firstly, we asked for general socio-demographic information on participants (e.g., age, gender) before collecting information about their experience with HRCs when selecting hospitals in the second part (e.g., Have you ever used one of the following German HRCs for referring patients into a specific hospital?). Thirdly, respondents were presented with publicly available hospital quality information items (see above) as well as short descriptions and were asked to rate each item on a 1–5 scale (1 = not all important; 5 = very important). Besides, we asked the respondents to select the single most important information item for the hospital referral decision. Afterwards, all referring physicians worked with HRCs as part of the survey to establish a basic understanding of them. In the fourth part, participating physicians were asked to respond to the UTAUT survey items (Table 1). The latter were measured on 1–7 scale (1 = strongly disagree; 7 = strongly agree).

For our study purpose, we purchased a database containing contact information (e.g., postal address, email) for 3,261 orthopedists in the German outpatient sector from a commercial provider (ArztData AG). This database covers about 88% of all orthopedists in the German outpatient sector (the total number of orthopedists in 2020 was reported to be 3,725 [43]). In the present study, we used a sequential mixed-mode strategy to achieve high response rates. In a first step, orthopedists were contacted via email, which contained a link to participate online (web-based survey). After one week, a first reminder was sent out. In a second step, the remaining orthopedists were contacted via mail and received a printed version of the survey; after two weeks, we sent out a second reminder. The questionnaire was piloted by 25 individuals to ensure the comprehensibility of the wording and internal validity; final adjustments were made accordingly. The survey was conducted between March and May 2021 in German language. As an incentive, respondents received a payment of 50€.

### Randomization

We used block randomization with randomly selected block sizes (i.e., 2, 4, and 6) to ensure balance in the allocation of participants between both study groups. By using a parallel group trial design, each orthopedist was randomly assigned to one of two study arms with equal probability [44]; i.e., to work with HRCs in the presence (intervention) or absence (control) of PROs. Participants were blinded to which group they were assigned to since they were not aware of the two different versions of the questionnaire (i.e., with/without PROs).

### Sample size calculation

The sample size calculation was based on Westland and colleagues [45] and determined 322 participants in total; i.e., 161 participants for each study arm, respectively. Therefore, the following assumptions were used: number of latent variables = 6, number of items = 30, effect size = 0.3 (the effect size refers to the intention to use HRCs for hospital referring purposes), power = 0.8, and a significance level of 0.05 (two-sided).

### Data analysis

SmartPLS 3.0 was used to transform our research model (see above) into a structural equation model [46]. The reliability and validity of the measurement model was determined by using a partial least squares path-weighting scheme. We assured content validity of the survey items by selecting well-established items from previous research. As shown in Table 1, all item loadings were above the recommended cutoff of 0.7 indicating item reliability [47]. The results for Cronbach’s $\alpha$ [48] and the composite reliability measure [49] of all constructs were above the recommended threshold of 0.7, indicating construct reliability of its items (see Supplementary Material 4 for more statistical details of the research model). For the structural research model, path coefficients are interpreted as regression coefficients using bootstrapping, a non-parametric technique for estimating the precision of the PLS estimates [50].

We extended the research model (see above) by employing a moderation mechanism to assess the effect of displaying PROs on HRC use intention [40, 51]. We included a binary moderating variable indicating the presence or absence of PROs on the HRC (yes/no). For each main independent variable, we determined whether or not we could detect statistically significant differences between the two study arms by conducting multigroup analysis [51]. In case of significant differences, we extended the original model by adding a moderating influence. Results are presented as both means and standard deviations for scaled survey items and as numbers and percentages for non-scaled survey items. To detect differences between the study arms, we applied chi-square tests (two-tailed test) and t-tests.

## Results

Overall, 591 referring orthopedists participated in our study and returned the survey (18.1% response rate; see Supplementary Material 5 for the CONSORT 2010 Flow Diagram). The following analysis reports on the 420 orthopedists who fully completed the UTAUT part of the questionnaire and provided consistent responses (Table 2). The mean age of all participating orthopedists was 53.48 (SD 8.04) years, a large majority of all respondents were male (88.8%), and 88 respondents (21.1%) indicated prior HRC experience to search for a hospital. As shown, we included slightly more respondents from the intervention group (*n* = 233; 55.5%) than from the control group (*n* = 187; 44.5%). Both groups were structurally equivalent, we did not detect significant differences between both study groups (*p* > 0.05 each). Besides, 248 respondents (59.0%) participated by answering our web-based online survey while 172 respondents (41.0%) filled in the paper and pencil survey. Again, we did not detect significant differences between both groups according to the survey mode (*p* > 0.05 each).

**Table 2:** Participant demographics: key characteristics (*p* value was calculated using Chi-square test and Welch *t* test)

	Study sample (*N* = 420)		No PROs		With PROs		*p*
			(*N* = 187; 44.5%)		(*N* = 233; 55.5%)		
	*N*	Mean (SD)/proportion	*N*	Mean (SD)/proportion	*N*	Mean (SD)/proportion	
Age							
Mean (SD)	416	53.48 (8.037)	186	53.70 (8.094)	230	53.29 (8.003)	.603
Gender	420		187		233		
Male	373	88.8%	169	90.4%	204	87.6%	.362
Female	47	11.2%	18	9.6%	29	12.4%	
Prior experience with HRC	417		184		233		
Yes	88	21.1%	39	21.2%	49	21.0%	.967
No	329	78.9%	145	78.8%	184	79.0%	
Perceived differences in the hospital quality	419		187		232		
No differences	3	0.7%	2	1.1%	1	0.4%	.662
Small differences	103	24.6%	50	26.7%	53	22.8%	
Big differences	309	73.7%	133	71.1%	176	75.9%	
I don’t know	4	1.0%	2	1.1%	2	0.9%	
Practice type	417		187		230		.315
Single physician practice	165	39.6%	65	34.8%	100	43.5%	
Group physician practice	166	39.8%	76	40.6%	90	39.1%	
Outpatient-based health-care centre	73	17.5%	40	21.4%	33	14.3%	
Other	13	3.1%	6	3.2%	7	3.0%	
Attending doctor (i.e., with hospital affiliation)							
Yes and I perform the surgery myself	85	20.4%	40	21.6%	45	19.4%	.792
Yes but I do not perform the surgery myself	51	12.2%	21	11.4%	30	12.9%	
No	281	67.4%	124	67.0%	157	67.7%	

In Table 3, we present the results regarding the first research question (RQ 1) of this study (i.e., the effect of presenting PROs on HRCs on the HRC use intention) from our UTAUT-based research model. As shown, the research model explains 58.0 percent of the variance in the dependent variable (*p* < 0.001). Based on our analysis, the presence of PROs was associated with an increased intention to use HRCs for hospital referral decision; however, the effect was not proven to be statistically significant (path coefficient: 0.033, *p* = 0.316).

**Table 3:** Effects on HRC intention to use [Study sample (*N* = 420)]

	Path coefficient ß	95% BCa CI^$^		*p* value
Main effects				
Attitude	0.116	− 0.014	0.235	0.076
Effort expectancy	− 0.051	− 0.134	0.022	0.195
Facilitating conditions	0.222	0.101	0.360	0.001
Performance expectancy	0.387	0.256	0.523	< 0.001
Social influence	0.122	0.047	0.203	0.002
Age	0.016	− 0.048	0.084	0.637
Experience	− 0.061	− 0.124	0.009	0.074
Gender	0.017	− 0.054	0.075	0.605
PROs display (yes/no)	0.033	− 0.028	0.101	0.316
Control variables				
Attending doctor	0.005	− 0.058	0.071	0.881
Hospital quality difference perception	− 0.040	− 0.096	0.016	0.148
Moderating effects				
Age × effort expectancy	− 0.002	− 0.069	0.098	0.968
Age × facilitating conditions	0.042	− 0.077	0.146	0.456
Age × performance expectancy	0.045	− 0.098	0.160	0.494
Age × social influence	0.058	− 0.087	0.156	0.332
Experience × effort expectancy	− 0.036	− 0.115	0.051	0.394
Experience × facilitating conditions	− 0.030	− 0.090	0.038	0.365
Experience × social influence	0.022	− 0.067	0.094	0.605
Gender × effort expectancy	0.025	− 0.079	0.120	0.619
Gender × facilitating conditions	0.034	− 0.082	0.148	0.564
Gender × performance expectancy	− 0.062	− 0.169	0.094	0.324
PROs × facilitating conditions	− 0.020	− 0.134	0.095	0.738
PROs × performance expectancy	0.047	− 0.067	0.166	0.419
*R*^2^ (in percent)	58.0 (*p* < .001)			

^$^Bootstrapping results (*N* = 5000) for bias corrected confidence intervals

In the following, we present the results regarding the second research question (RQ 2) of this study. First, Table 3 also displays the results regarding the determinants influencing participating orthopedists to use HRCs. Based on our analysis, three of the five main constructs could be shown to be significantly related to HRC use intention. Most importantly, performance expectancy (path coefficient: 0.387, *p* < 0.001), facilitating conditions (path coefficient: 0.222, *p* < 0.05), and social influence (path coefficient: 0.122, *p* < 0.05) are significantly and positively related to HRC use intention. The two other main UTAUT variables (attitude and effort expectancy) showed no significant effects on the intention to use HRC. In more detail, performance expectancy was the only main independent variable that was shown to be significantly and positively related to HRC use intention in both groups. In addition, attitude was significantly and positively related to HRC use intention in the control group, while facilitating conditions and social influence were also significantly and positively related to HRC use intention in the intervention group, respectively [Supplementary Material 6].

Next, Table 4 presents the importance of both traditional quality measures as well as PROs for the hospital referral decision (RQ 3). Across both study arms, the results appear to be very consistent. On a 1–5 scale (1 = not at all important; 5 = extremely important), referring orthopedists rated “complication rate” (4.19 ± 1.06; 4.11 ± 1.03), “the number of cases treated” (4.09 ± 0.98; 4.07 ± 1.04), and “1-year revision surgery rate” (3.98 ± 1.22; 3.93 ± 1.20) as most important. Participating orthopedists from the intervention arm rated “PROs” slightly less important (3.84 ± 1.09). In addition, the survey results for the single most important information item for the hospital referral choice confirmed “the number of cases treated” (34.3%; 32.1%) and “complication rate” (25.2%; 22.2%) as most important in both study arms. In the intervention study arm, “PROs” were selected by 20.9% of all participants as the single most important information.

**Table 4:** Importance of different information items for the hospital referral decision (*p* value was calculated using *t* tests; *N* = 420)

Information items for the hospital referral decision	Importance of different information items [Mean (SD)]*			Most important information item^$^	
	No PROs (*N* = 187; 44.5%)	With PROs (*N* = 233; 55.5%)	*p*	No PROs (*N* = 187; 44.5%)	With PROs (*N* = 233; 55.5%)
1-year revision surgery rate^1^	3.98 (1.22)	3.93 (1.20)	.630	16.0%	13.0%
Complication rate^2^	4.19 (1.06)	4.11 (1.03)	.434	25.2%	22.2%
Mobility at hospital discharge^3^	3.29 (1.17)	n.a	n.a	12.6%	n.a
The number of cases treated^4^	4.09 (0.98)	4.07 (1.04)	.816	34.3%	32.1%
Confirmed diagnosis (hip surgery) rate^5^	3.69 (1.23)	3.81 (1.12)	.297	6.6%	6.1%
Endocert certificate^6^	3.06 (1.16)	3.15 (1.23)	.461	5.3%	5.7%
Patient reported outcomes (PROs)	n.a	3.84 (1.09)	n.a	n.a	20.9%

*importance of items is rated on a 1–5 scale [1 not all important; 5 extremely important]

^$^which information was most important for your hospital choice?

^1^percentage of patients who underwent revision surgery within one year after initial surgery

^2^postoperative specific complications after hip replacement surgery (e.g., infections, bleeding, thromboembolism)

^3^functional mobility status on discharge after hip replacement surgery

^4^the number of total elective hip replacement surgeries in a hospital

^5^hip replacement primary implantation with fulfilled indication criteria

^6^hospitals which meet requirements of the EndoCert certification system

The descriptive survey results for all UTAUT items (Table 5) can provide a more in-depth picture of what orthopedists from the German outpatient sector think of using HRCs for referral purposes (RQ 4). For example, 45% resp. 49% of all responding orthopedists stated they were open to using HRCs for hospital referral purposes and 43% resp. 44% agreed with the statement that using HRCs for referring patients into a hospital is a good idea. Furthermore, the ease of using HRC does not seem to be a concern among referring orthopedists; for example, approximately 70% of all respondents agreed with the statement that is easy to learn how to use HRCs. In contrast, HRCs do not seem to fit well with the way most orthopedists like to search for a hospital; here, 51% resp. 43% did not agree with this statement. The results for performance expectancy-related items show that referring physicians seem to support the usefulness of HRCs to search for a hospital in general; here, 42% resp. 44% agreed with this statement. However, the majority of all respondents seem to have doubts whether HRCs can help them find the best hospital. Social influence-related items could not be shown to be supported among referring physicians. For example, 69% resp. 65% of all respondents would not feel uncomfortable if their colleagues used HRCs but they did not. Finally, respondents are more likely not to use HRCs for the next hospital referral of their patients; here, only 16% resp. 26% supported the HRC use intention for the next hospital search. Regarding the latter, the use intention was significantly higher in the presence of PROs on HRCs (*p* = 0.035).

**Table 5:** Descriptive survey results for all UTAUT items (based on a rating on a 1–7 Scale with 1 strongly disagree and 7 strongly agree) (percentages; equals 420 finisher) (*p* value was calculated using Chi-square test)

Construct	ID	Item	No PROs (*N* = 187; 44.5%)			With PROs (*N* = 233; 55.5%)			*p*
			Disagree^§^	Neutral^$^	Agree^&^	Disagree^§^	Neutral^$^	Agree^&^	
Attitude (AT)	AT1	Using hospital report cards for referring patients into a hospital is a good idea	31.6	25.1	43.3	30.5	25.8	43.8	.971
	AT2	Hospital report cards makes searching for a hospital more interesting	28.9	24.6	46.5	27.9	19.7	52.4	.394
	AT3	I like working with hospital report cards	65.2	22.5	12.3	54.7	25.9	19.4	.060
	AT4	Working with hospital report cards is fun	56.1	36.4	7.5	51.5	36.1	12.4	.233
	AT5	I am open for using hospital report cards to search for a hospital	28.9	26.2	44.9	21.9	29.6	48.5	.254
Effort expectancy (EE)	EE1	My interaction with hospital report cards is clear and understandable	14.4	38.5	47.1	16.3	32.2	51.5	.401
	EE2	It is easy for me to become skilful at using hospital report cards	22.5	36.4	41.2	21.0	28.8	50.2	.150
	EE3	Learning how to use hospital report cards is easy for me	3.2	27.8	69.0	5.6	21.6	72.8	.203
	EE4	I find hospital report cards easy to use	4.8	39.0	56.1	8.2	26.6	65.2	.017
Facilitating conditions (FC)	FC1	I have the resources necessary to use hospital report cards (e.g., internet connection).*	1.6	3.2	95.2	2.1	5.6	92.3	.462
	FC2	I have the knowledge necessary to use hospital report cards.*	2.7	7.0	90.4	3.0	9.0	88.0	.722
	FC3	Hospital report cards are compatible with other information sources (e.g., opinion of colleagues) that I use to search for a hospital	21.4	27.8	50.8	17.6	25.3	57.1	.413
	FC4	I think that using hospital report cards fits well with the way I like to search for a hospital	51.3	25.1	23.5	42.5	26.2	31.3	.131
Performance expectancy (PE)	PE1	I find hospital report cards useful to search for a hospital for my patients	35.8	22.5	41.7	33.9	21.9	44.2	.871
	PE2	Using hospital report cards enables me to search for a hospital more quickly	49.2	27.3	23.5	38.6	35.2	26.2	.081
	PE3	Using hospital report cards helps me find the best hospital	47.1	25.1	27.8	41.2	24.9	33.9	.359
	PE4	If I use hospital report cards, I will increase the chances of my patients of feeling well after the surgery	41.7	26.2	32.1	35.6	24.0	40.3	.209
Social influence (SI)	SI1	People who influence my behaviour think that I should use hospital report cards	55.1	34.2	10.7	52.8	34.3	12.9	.773
	SI2	People who are important to me think that I should use hospital report cards	58.3	29.4	12.3	54.9	28.3	16.7	.442
	SI3	I would feel uncomfortable if my colleagues use hospital report cards and I do not	69.0	19.3	11.8	64.8	20.6	14.6	.610
	SI4	I would feel uncomfortable if my patients use hospital report cards and I do not	67.9	20.9	11.2	64.8	22.7	12.4	.799
Intention to use (IU)	IU1	I intend to use hospital report cards for the next hospital referral of my patients	62.4	21.5	16.1	56.3	17.3	26.4	.035

^§^the percentage of respondents who stated to disagree at least to some extent to the stated UTAUT items

^$^the percentage of respondents who stated neither to agree nor disagree to the stated UTAUT items

^&^the percentage of respondents who stated to agree at least to some extent to the stated UTAUT items

*based on the instrument validation process, item was removed

## Discussion

The aim of this study was to determine the intention to use HRCs for hospital referral purposes in the presence or absence of PROs as well as to explore the relevance of publicly available hospital performance information from the perspective of referring physicians. Therefore, we surveyed a sample of 420 referring orthopedists for hip replacement surgery from the German outpatient sector. Although the present study was conducted in Germany, the results presented in this paper are of interest to all countries with public reporting initiatives, such as the United States, England, and others.

Our study sample seems to be representative of orthopedists in the German outpatient sector. For example, the mean age of all 420 surveyed physicians in our study was 53.5 years, which is very similar to the mean age of all orthopedists in the German outpatient sector (54.2 in 2020) [52]. Also, the percentage of female participants in our study (11.2%) was quite similar to the percentage of female referring physicians among all German orthopedists in 2020 (14.6%) [52]. Regarding the practice type, our study sample comprised a higher percentage of referring physicians from single practices (39.6% vs. 31.9%), whereas physicians from outpatient-based healthcare centers were underrepresented (17.5% vs. 29.1%) [52].

First of all, the present study showed that presenting PROs on HRCs was not associated with an increased use intention of HRCs for the next hospital search from the perspective of referring physicians. This is in line with a similar study from Germany which also demonstrates that the presence of PROs on HRCs was not associated with an increased HRCs use intention to among 447 insurees from a German statutory health insurance who had undergone elective hip arthroplasty surgery. [34] Further studies should continue to investigate whether and especially how to present PROs on HRCs. It is possible that PROs were not presented in a comprehensible and meaningful way in our study. We labeled PROs “Results from the patient’s perspective” and placed an explanatory description of PROs before answering the choice tasks. However, as physicians might not be familiar with PROs, some might have confused PROs with traditional hospital-related patient satisfaction measures and therefore assigned them less relevance. Therefore, we ask readers to treat our findings as exploratory rather than definitive, since this is the first explanatory investigation within this complex area of hospital referral purpose behavior. Currently, however, it seems to be the case that the presentation of interesting and valuable information—such as PROs—on HRCs as a single approach is not likely to increase the HRC use intention.

The present study is the first in-depth investigation exploring the determinants of the intention to use HRCs from the perspective of referring physicians. Most of the available literature on the impact of publicly reported quality information from the perspective of referring physicians has followed a descriptive approach (see above) [14, 21–25]. As shown, the applied research model explains 58% of the variance in the dependent variable; the explanatory power of our models is very similar to the two related studies in this context [33, 34] and higher than other studies investigating the information technology use intention in healthcare [33]. The findings indicate the dominant role of “performance expectancy” for explaining the intention to use HRC, which was shown to be significantly and positively related to HRC use intention overall and in both subsamples. This finding underpins the purpose of HRCs, namely to support referring physicians in searching for a hospital in general [15, 53] and is mostly in line with other studies in healthcare settings and a study that explains the use of HRCs among current users and non-users in the US [33]. Based on our results, it seems that around 70% of referring physicians could be—at least to some extent—responsive to publicly reported hospital quality information. Those might see both the need and potential benefit of HRCs for supporting them in the hospital referral process [25]. This is also in accordance with our finding that 74% of all surveyed referring orthopedists stated that they perceived substantial differences in the quality of care between hospitals. However, the majority of all respondents seem to have doubts as to whether HRCs can help them find the best hospital. For example, most referring orthopedists did not agree with the statement that HRCs enable them to search for a hospital more quickly. So far, only 16% resp. 26% supported HRC use intention for the next hospital search in its current form. In contrast, patients have a much more positive attitude towards using HRCs for the hospital choice. Here, about two-thirds (64.6% resp. 68.9%) of the 447 surveyed patients confirmed the intention to use HRCs the next time they searched for a hospital [34].

The findings from this study present consistent results on the importance of publicly available hospital-quality information for the hospital referral decision. We could see that referring orthopedists rated “complication rate”, “the number of cases treated”, and “1-year revision surgery rate” as most important; in contrast, “PROs” were rated slightly less important. These results are somewhat contradictory to those from the patients' perspective; here, PROs were rated as most important according to both the rating-based as well as ranking-based results. For example, PROs were rated most frequently as the single most important information item for the hospital choice among patients (37.6%) followed by “confirmed diagnosis (hip surgery) rate” (24.8%) [34]. In a previously published study on a sample of 300 German outpatient-based physicians, referring physicians valued most information that reflects their own and their patients’ experiences with a hospital, the hospital’s expertise as well as the results of treatment [15]. This is mostly in line with our findings which have shown a dominance of effectiveness-related (“complication rate”, “1-year revision surgery rate”) and experience-related (“the number of cases treated”) measures. Another study has also confirmed the “frequency of relevant procedures” as one very important hospital-quality criteria [54]. In contrast, patient-experience measures (e.g., whether or not patients were treated in a friendly and respectful manner) as well as physician-experience measures were excluded in our preliminary qualitative research; the latter due to the non-availability for public reporting purposes. Geraedts and colleagues have also shown that hospitals’ structural characteristics play a minor role from the perspective of referring physicians confirming our findings [15]. For example, whether or not a hospital was successfully certified as an EndoProsthetics Center was rated least important in our study.

In this context, our study adds to the literature by investigating the specific role of PROs for the hospital referral decision. We showed that PROs were rated slightly less important than effectiveness and experience-related measures (see above), but were also considered an important information criterion for the hospital choice. For example, one in five surveyed referring orthopedist selected “PROs” as the single most important information. There is hardly any evidence available from other studies with a comparable research question. For example, the above-mentioned study did not include “PROs” as a quality measure to be rated by surveyed physicians [15]. Furthermore, the available evidence on the importance of PROs for choosing a hospital was mainly conducted from the perspective of patients [37, 55, 56]. One reason for the relatively low number of studies with a special focus on the role of PROs as an information item for choosing a hospital might be due to the fact that the collection and reporting of PROs is still very much in its early stages [27, 57].

Another important finding which can be derived from our analysis is the fact that most referring physicians (80%) still not have used publicly reported hospital quality information or HRCs for referral purposes. This is in line with the study by Ketelaar and colleagues, showing that only 12% of surveyed GPs in the Netherlands reported to have used such quality information measures for selecting a hospital [25]. This implies a large potential of HRCs to provide quality information to more referring physicians in case of higher awareness and usage rates in the near future. In this context, we should consider that evidence from the United States has identified unfulfilled expectations among HRC users [33]. This result has pointed to a certain frustration or disillusion among HRC users and indicated that using existing report cards might not be as useful as assumed to find an appropriate healthcare provider. A similar conclusion might be derived from our analysis since only 16% supported HRC use intention for the next hospital search in its current form. However, we should bear in mind that we did not include HRCs in its current forms but presented the hospital quality information in a more neutral style.

Our findings should be considered in light of some limitations. Firstly, our study was conducted in the German healthcare setting and might be of limited relevance for other countries. Secondly, regarding the representativeness of our study sample, we did not obtain information regarding other interesting variables; for example, we were not able to evaluate the regional distribution of our sample. Thirdly, we conducted a comprehensive preliminary study to determine the most relevant quality information measures from the perspective of referring physicians. However, no German HRC contains the quality information in the presented form. Most HRCs publicly report on additional or alternative measures and have applied different presentation forms, making generalizations to any real-world site challenging [58]. Therefore, we urge readers to be careful when implementing our findings into practice or further research. Fourth, in the present study, we aimed to add to the current discussion by using a novel approach to learn more about the effect of presenting PROs on HRCs on the use intention of HRCs as well as the determinants influencing referring physicians to use HRCs as well. In the present paper, we draw on the UTAUT acceptance model and used structural equation modeling for our analysis. It is important to mention that other techniques could also have been applied and might have led to different findings. Furthermore, we used a sequential mixed-mode strategy (online survey with a reminder followed by a paper survey with a reminder) in order to achieve high response rates. As stated above, we did not find any significant differences between those respondents who answered our web-based online survey and those respondents who filled in the paper and pencil survey. However, it should be mentioned that we detected slightly different responses for 3 out of the 21 UTAUT-related items. In two cases (EE2), web-based survey respondents gave more favorable results. In contrast, paper-based survey respondents gave more favorable results regarding two further items (EE1, PE4). Next, it is important to mention that a considerable number of respondents were excluded from our final. Unfortunately, we are not able to present a detailed and meaningful comparison of both groups since several respondents did not provide socio-demographic information, etc. So, we cannot determine whether there are both known and unknown differences between both groups. Again, we ask the reader to take this limitation into account when interpreting the results of this study. Finally, our research was based on the original version of the UTAUT model [31]. It should be noted, that Venkatesh and colleagues have published an extended version of the UTAUT model (i.e., the UTAUT2 model [59]) which has been used extensively so far [60]. Our decision to use the original UTAUT model was based on the fact that past research showed well-performing model results for a similar study aim [33], our doubts that the added constructs in the UTUAT2 model would lead to more helpful findings (e.g., we did not expect much explanatory value from responses to items such as “I am addicted to using [HRCs]” or “Using [HRCs] has become natural to me” since we know from previous research that referring physicians do not have much experience with HRCs), and the fact that we determined the value of other constructs. For example, aspects such as self-efficacy and information quality play an important role for novel IT use intention among physicians in general. We thus added those constructs to our model in a first step but could not see any explanatory value resulting from those.

In sum, this study adds to the literature by presenting results of a survey among 420 referring physicians from the German outpatient sector to assess HRC adoption for hospital referral purposes in the presence or absence of PROs. Our findings showed that presenting PROs on HRCs was not associated with an increased use intention of HRCs for the next hospital search. Nevertheless, the findings underpin the purpose of HRCs, namely to support referring physicians to search for a hospital in general [15, 53]. However, the majority of all respondents seem to have doubts as to whether HRCs can help them find the best hospital. We show that PROs were rated slightly less important than effectiveness-related and experience-related measures, but were also considered an important information criterion for the hospital choice. Further studies should continue to investigate whether and especially how to present PROs on HRCs as well as to investigate other strategies that might lead to higher intention rates.

### Supplementary Information

Below is the link to the electronic supplementary material.

Supplementary file1 (DOCX 691 KB)

## Data Availability

Data available on request from the corresponding author (martin.emmert@uni-bayreuth.de).
