# Supplementary material for: Referring physicians' intention to use hospital report cards for hospital referral purposes in the presence or absence of patient-reported outcomes: a randomized trial
Source: Eur J Health Econ. 2023 Apr 13;25(2):293–305. doi: 10.1007/s10198-023-01587-6 (PMC10858825; doi:10.1007/s10198-023-01587-6)
Supplement: Supplementary file 1 — Supplementary file1 (DOCX 691 KB) [file 10198_2023_1587_MOESM1_ESM.docx]

Supplementary Material

*Supplementary Material 1.* PRISMA 2020 flow diagram regarding the preliminary systematic literature review


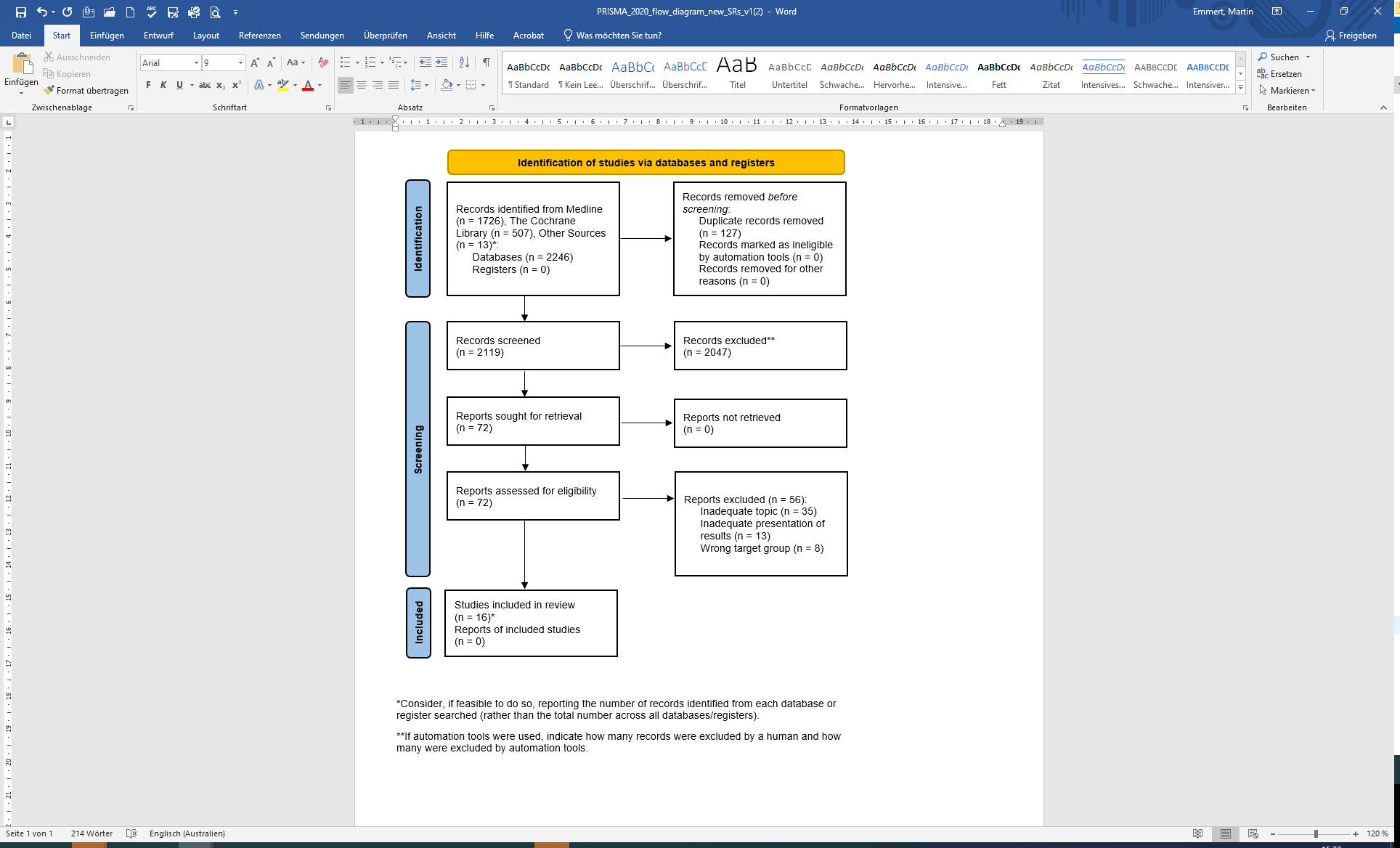


*Supplementary Material 2.* Overview of relevant criteria for referring patients into hospitals from the perspective of referring physicians (literature-based results)

| Nr | Criteria for hospital referral decision making | Relevance (literature-based) |
| --- | --- | --- |
| 1 | Patient travel distance to the hospital | Middle |
| 2 | Distance from physician’s practice to the hospital | Low |
| 3 | Teaching hospital | Low |
| 4 | Further medical training (hospital doctors) | Middle |
| 5 | Hygiene measures resp. infection rates | Middle |
| 6 | Medical equipment | Middle |
| 7 | The number of cases treated | High |
| 8 | Range of medical services | Middle |
| 9 | Number of specialists | Middle |
| 10 | Endocert Certificate | Middle |
| 11 | Mortality rate | Low |
| 12 | Complication rate | High |
| 13 | Indirect Complication rate | Middle |
| 14 | Confirmed diagnosis (hip surgery) rate | Middle |
| 15 | Prevention of falls measures | Middle |
| 16 | Mobility at hospital discharge | Middle |
| 17 | Ability to walk at hospital discharge | Middle |
| 18 | 1-year revision surgery rate | Middle |
| 19 | Patient recommendation | Low |
| *20* | *Patient reported outcomes (PROs)* | *n.a.* |

*Supplementary Material 3.* The research model

**
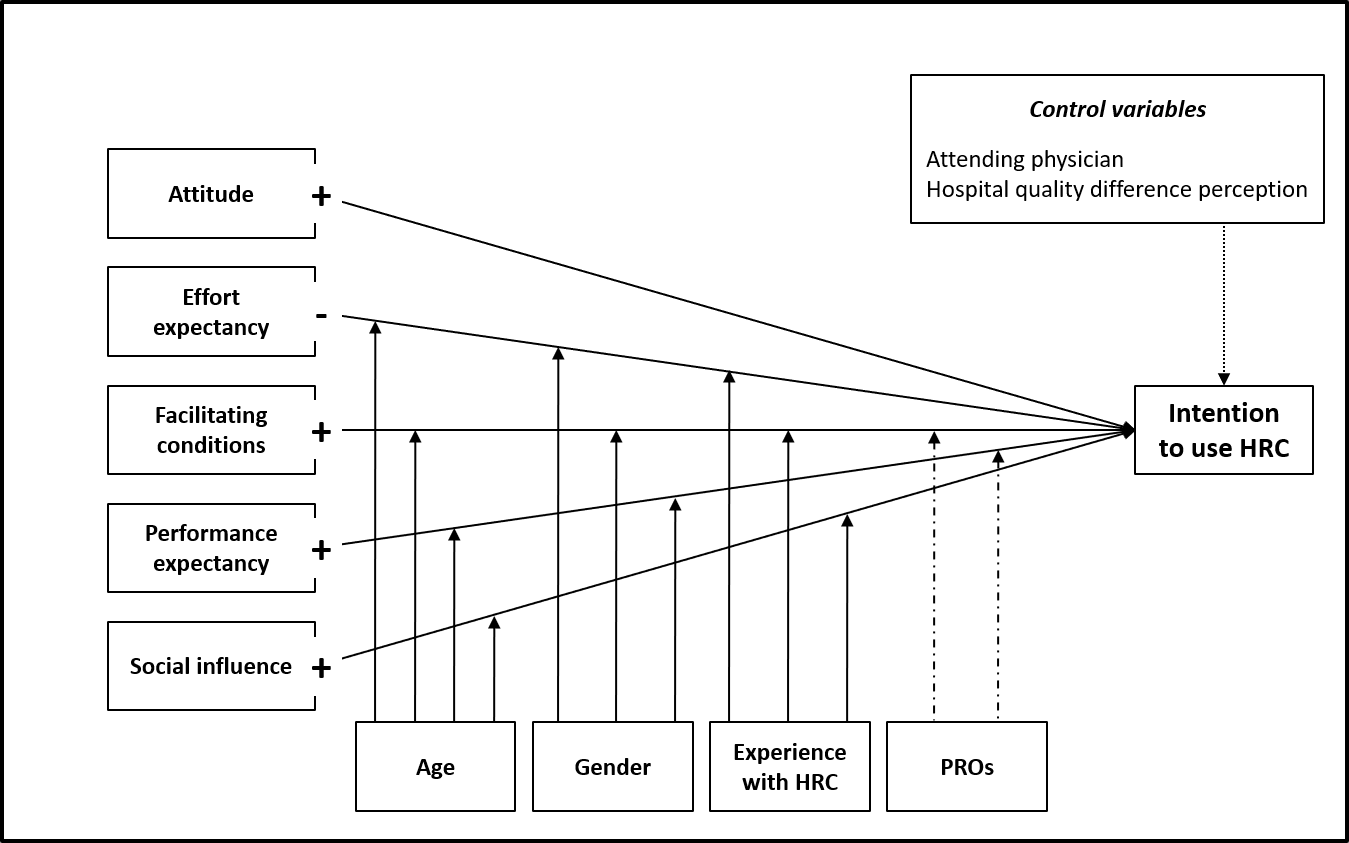
**

*Supplementary Material 4.* Correlations among main variables

|  | Age | Experience | Gender | PROs | AT | EE | FC | PE | SI | IU |
| --- | --- | --- | --- | --- | --- | --- | --- | --- | --- | --- |
| Age | 1.000 | -0.017 | -0.170 | -0.025 | -0.099 | -0.126 | -0.069 | -0.032 | -0.054 | -0.002 |
| Experience | -0.017 | 1.000 | -0.020 | 0.002 | 0.037 | 0.081 | 0.051 | 0.028 | -0.024 | -0.035 |
| Gender | -0.170 | -0.020 | 1.000 | 0.044 | 0.085 | -0.015 | 0.056 | 0.051 | 0.101 | 0.053 |
| PROs display (yes/no) | -0.025 | 0.002 | 0.044 | 1.000 | 0.077 | 0.047 | 0.085 | 0.068 | 0.057 | 0.090 |
| Attitude (AT) | -0.099 | 0.037 | 0.085 | 0.077 | 1.000 | 0.487 | 0.792 | 0.813 | 0.502 | 0.647 |
| Effort expectancy (EE) | -0.126 | 0.081 | -0.015 | 0.047 | 0.487 | 1.000 | 0.438 | 0.427 | 0.179 | 0.281 |
| Facilitating conditions (FC) | -0.069 | 0.051 | 0.056 | 0.085 | 0.792 | 0.438 | 1.000 | 0.801 | 0.480 | 0.668 |
| Performance expectancy (PE) | -0.032 | 0.028 | 0.051 | 0.068 | 0.813 | 0.427 | 0.801 | 1.000 | 0.536 | 0.708 |
| Social influence (SI) | -0.054 | -0.024 | 0.101 | 0.057 | 0.502 | 0.179 | 0.480 | 0.536 | 1.000 | 0.497 |
| Intention to use (IU) | -0.002 | -0.035 | 0.053 | 0.090 | 0.647 | 0.281 | 0.668 | 0.708 | 0.497 | 1.000 |
| Cronbach’s alpha | 1.000 | 1.000 | 1.000 | 1.000 | 0.873 | 0.818 | 0.821 | 0.923 | 0.890 | 1.000 |
| Composite reliability | 1.000 | 1.000 | 1.000 | 1.000 | 0.908 | 0.877 | 0.917 | 0.946 | 0.924 | 1.000 |
| AVE | 1.000 | 1.000 | 1.000 | 1.000 | 0.665 | 0.642 | 0.848 | 0.813 | 0.751 | 1.000 |

*Supplementary Material 5.* CONSORT 2010 Flow Diagram

**
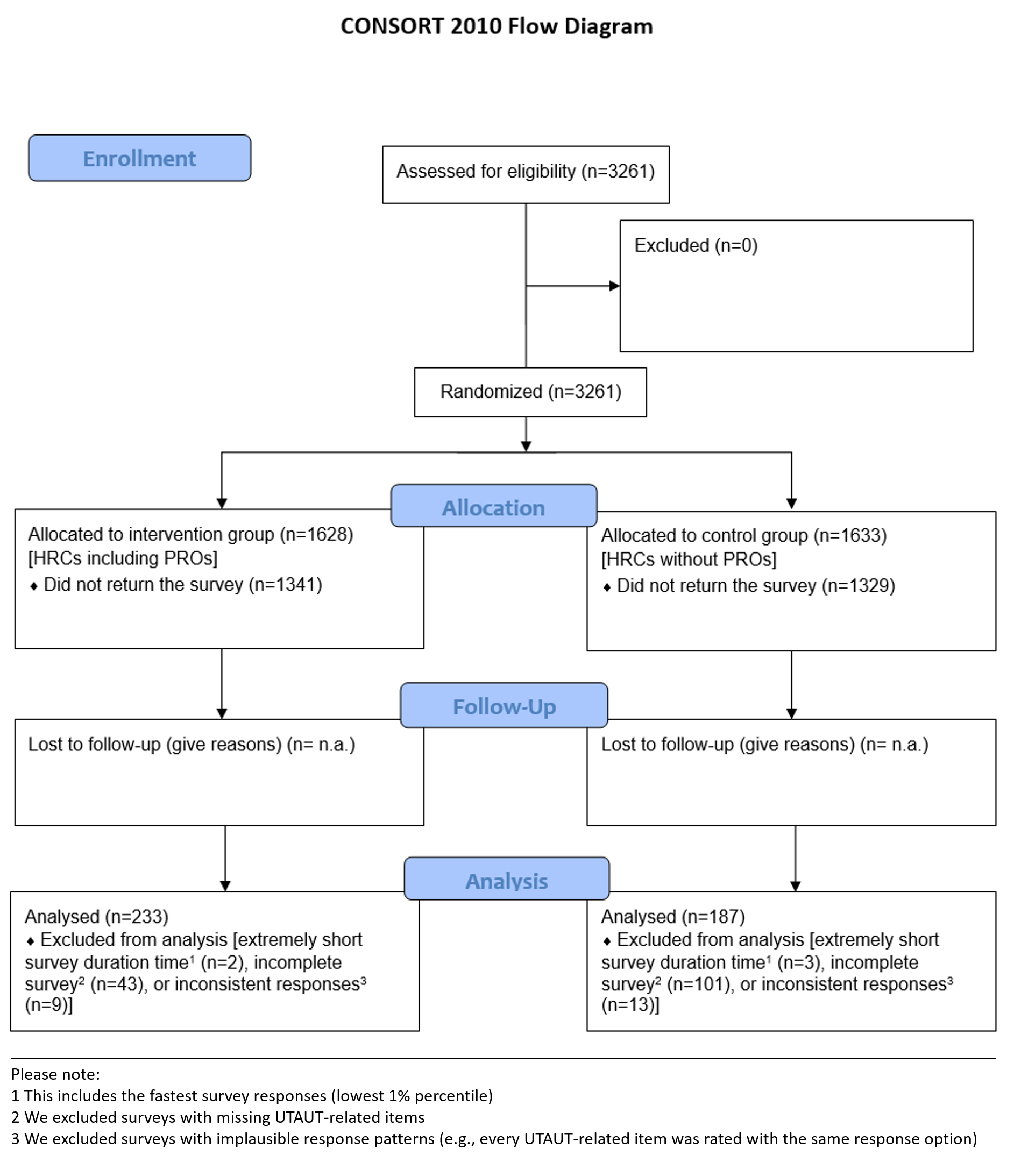
**

*Supplementary Material 6.* Effects on HRC intention to use; multi-group analysis (without PROs vs. with PROs)

|  | No PROs  (N=187; 44.5%) | | | | With PROs (N=233; 55.5%) | | | |
| --- | --- | --- | --- | --- | --- | --- | --- | --- |
|  | *Path coefficient ß* | *95% BCa CI^$^* | | *p-value* | *Path coefficient ß* | *95% BCa CI^$^* | | *p-value* |
| Main effects |  |  |  |  |  |  |  |  |
| Attitude | 0.309 | 0.121 | 0.495 | 0.001 | 0.009 | -0.164 | 0.175 | 0.914 |
| Effort expectancy | -0.065 | -0.165 | 0.054 | 0.257 | -0.049 | -0.174 | 0.039 | 0.363 |
| Facilitating conditions | 0.192 | -0.022 | 0.398 | 0.073 | 0.224 | 0.060 | 0.402 | 0.013 |
| Performance expectancy | 0.249 | 0.056 | 0.489 | 0.022 | 0.466 | 0.281 | 0.645 | <0.001 |
| Social influence | 0.093 | -0.045 | 0.227 | 0.183 | 0.131 | 0.021 | 0.236 | 0.016 |
| Age | 0.025 | -0.083 | 0.123 | 0.641 | -0.017 | -0.109 | 0.071 | 0.702 |
| Experience | -0.088 | -0.223 | 0.004 | 0.112 | -0.044 | -0.139 | 0.058 | 0.378 |
| Gender | -0.018 | -0.173 | 0.101 | 0.803 | 0.020 | -0.057 | 0.100 | 0.617 |
| Control variables |  |  |  |  |  |  |  |  |
| Attending doctor (practitioner with hospital affiliation) | 0.030 | -0.057 | 0.135 | 0.551 | -0.019 | -0.107 | 0.069 | 0.673 |
| Hospital quality difference perception | -0.045 | -0.129 | 0.045 | 0.296 | -0.035 | -0.117 | 0.035 | 0.360 |
| Moderating effects |  |  |  |  |  |  |  |  |
| Age x Effort expectancy | 0.027 | -0.064 | 0.169 | 0.643 | -0.043 | -0.191 | 0.093 | 0.566 |
| Age x Facilitating conditions | 0.023 | -0.179 | 0.242 | 0.826 | 0.033 | -0.116 | 0.170 | 0.651 |
| Age x Performance expectancy | 0.095 | -0.157 | 0.315 | 0.428 | 0.046 | -0.103 | 0.220 | 0.590 |
| Age x Social influence | 0.083 | -0.079 | 0.259 | 0.330 | 0.062 | -0.041 | 0.218 | 0.427 |
| Experience x Effort expectancy | 0.027 | -0.118 | 0.119 | 0.633 | -0.062 | -0.177 | 0.057 | 0.297 |
| Experience x Facilitating conditions | 0.060 | -0.060 | 0.206 | 0.393 | -0.056 | -0.158 | 0.051 | 0.292 |
| Experience x Social influence | 0.037 | -0.104 | 0.151 | 0.570 | -0.052 | -0.198 | 0.019 | 0.452 |
| Gender x Effort expectancy | 0.113 | -0.051 | 0.293 | 0.214 | -0.023 | -0.152 | 0.100 | 0.725 |
| Gender x Facilitating conditions | 0.064 | -0.149 | 0.316 | 0.584 | 0.038 | -0.070 | 0.209 | 0.598 |
| Gender x Performance expectancy | -0.172 | -0.473 | 0.044 | 0.163 | -0.040 | -0.201 | 0.102 | 0.612 |
| R^2^ (in percent) | 63.0 | | | | 58.9 | | | |
| ^$^ Bootstrapping results (N=5000) for Bias Corrected Confidence Intervals | | | | | | | | |
